# The combinatory effect of sinusoidal electromagnetic field and VEGF promotes osteogenesis and angiogenesis of mesenchymal stem cell-laden PCL/HA implants in a rat subcritical cranial defect

**DOI:** 10.1186/s13287-019-1464-x

**Published:** 2019-12-16

**Authors:** Jingyuan Chen, Chang Tu, Xiangyu Tang, Hao Li, Jiyuan Yan, Yongzhuang Ma, Hua Wu, Chaoxu Liu

**Affiliations:** 1 Department of Orthopedics, Tongji Hospital, Tongji Medical College, Huazhong University of Science and Technology, Jiefang Avenue 1095, Wuhan, 430030 China; 2 Department of Radiology, Tongji Hospital, Tongji Medical College, Huazhong University of Science and Technology, Jiefang Avenue 1095, Wuhan, 430030 China

**Keywords:** Bone tissue engineering, Osteogenesis, Vascularization, Sinusoidal electromagnetic fields, Mesenchymal stem cell

## Abstract

**Background:**

Restoration of massive bone defects remains a huge challenge for orthopedic surgeons. Insufficient vascularization and slow bone regeneration limited the application of tissue engineering in bone defect. The effect of electromagnetic field (EMF) on bone defect has been reported for many years. However, sinusoidal EMF (SEMF) combined with tissue engineering in bone regeneration remains poorly investigated.

**Methods:**

In the present study, we investigated the effect of SEMF and vascular endothelial growth factor (VEGF) on osteogenic and vasculogenic differentiation of rat bone marrow-derived mesenchymal stem cells (rBMSCs). Furthermore, pretreated rBMSC- laden polycaprolactone-hydroxyapatite (PCL/HA) scaffold was constructed and implanted into the subcritical cranial defect of rats. The bone formation and vascularization were evaluated 4 and 12 weeks after implantation.

**Results:**

It was shown that SEMF and VEGF could enhance the protein and mRNA expression levels of osteoblast- and endothelial cell-related markers, respectively. The combinatory effect of SEMF and VEGF slightly promoted the angiogenic differentiation of rBMSCs. The proteins of Wnt1, low-density lipoprotein receptor-related protein 6 (LRP-6), and β-catenin increased in all inducted groups, especially in SEMF + VEGF group. The results indicated that Wnt/β-catenin pathway might participate in the osteogenic and angiogenic differentiation of rBMSCs. Histological evaluation and reconstructed 3D graphs revealed that tissue-engineered constructs significantly promoted the new bone formation and angiogenesis compared to other groups.

**Conclusion:**

The combinatory effect of SEMF and VEGF raised an efficient approach to enhance the osteogenesis and vascularization of tissue-engineered constructs, which provided a useful guide for regeneration of bone defects.

## Background

Restoration of massive bone defects resulted from significant trauma, tumor excision, inflammation, osteoporosis, or pathological fractures remains an unfulfilled challenge for orthopedic surgeons [1]. Bone grafts remain as the current crucial therapeutic intervention for these massive defects, e.g., autologous bone grafts, allogeneic bone grafts, xenogeneic bone grafts, and tissue engineering bone grafts [2]. Autologous grafts possess all the essential attributes: they are osteoinductive (bone morphogenetic proteins (BMPs) and other growth factors), osteoconductive (scaffold), and osteogenic (cells) [3, 4]. As a result, autogenous bone grafting remains the gold standard for bony defect repair. However, this approach is limited by numerous drawbacks, such as finite supply, donor site morbidity, pain, and risk of infection [5]. As an alternative, xeno/allografts are widely employed. Nevertheless, deficiencies, including host rejection, risk of disease transmission, high cost, lacking osteogenicity, and vascularization, limited the application of xeno/allografts in clinic [4]. By comparison, bone tissue engineering has shown great potentials in bone regeneration and attracted great interests in the past decades [6].

Bone tissue engineering consists of four fundamental components: cells, biomaterials or scaffolds, bioactive growth factors, and other stimulus [7]. Ideal scaffolds play a crucial role in offering a three-dimensional framework for the adhesion, proliferation, and differentiation of stem cells, which should be biocompatible, biodegradable, and osteoconductive and have satisfying mechanical properties [7, 8]. Recently, three-dimensional (3D) printing is widely employed to fabricate cell-laden scaffolds, which possess designed shapes, controlled chemistry, and interconnected porosity [9]. However, the low degree of neovascularization to meet the growing tissue nutrient supply and metabolic products clearance needs is still a huge challenge with bone tissue engineering [10].

Mesenchymal stem cells (MSCs), first reported by Friedenstein et al. [11], are multipotent adult stem cells and have been isolated from different tissues including bone marrow, adipose tissue, synovial tissue, umbilical cord blood, and peripheral blood [12, 13]. MSCs from various tissues are not identical and have differences in proliferation potential, differentiation ability, and regeneration function [14], while most of them possess the capacity of differentiation into numerous cell types like osteoblasts, chondrocytes, and adipocytes [15]. Furthermore, researchers have found various approaches to accelerate MSCs differentiating into endothelial-like cells, such as vascular endothelial cell growth factor (VEGF) [16], micro-topography [17], and mechanical stretch [18]. Thus, due to the multidirectional differentiation, MSCs become one of the extensively utilized stem cell resources for achieving the multifunctional properties of tissue-engineered bone substitutes.

Since Bassett et al. first used electromagnetic field (EMF) to induce osteogenesis in 1974 [19], the therapeutic effects of EMF on bone regeneration have been widely investigated in the past decades. Substantial and growing evidence has shown that pulsed EMF (PEMF) could inhibit bone loss, improve bone quality, and promote proliferation and mineralization of osteoblasts [20, 21]. PEMF could also facilitate osteogenic differentiation of MSCs, despite the various combinations of frequency, intensity, and exposure time in different studies [22, 23]. Therefore, numerous investigations about synergetic effect of PEMF and biomaterials (calcium phosphate, polymers, titanium) on bone defect regeneration have been published in recent years [24, 25]. Another type of EMF, sinusoidal electromagnetic fields (SEMF), could also enhance the bone repair and accelerate the osteogenesis differentiation of rat bone marrow-derived mesenchymal stem cells (rBMSCs) [26–29]. However, the incorporation of SEMF, MSCs, and bioscaffold to accelerate bone formation has not yet been reported.

Hence, in the present study, we seeded rBMSCs on polycaprolactone-hydroxyapatite (PCL/HA) composite scaffolds and inducted with VEGF or SEMF to construct a vascularized tissue-engineered bone grafts. We hypothesized that the stimulation of SEMF and VEGF on rBMSCs will synergistically and vigorously enhance the osteogenesis and vascularization in engineered bone constructs and facilitate the regeneration of bone defect. After evaluating the osteogenic and endothelial differentiation through related markers, we implanted the pretreated engineered bone constructs into subcritical cranial bone defects of rats to investigate the vascularization and osteogenesis efficacy of the constructs.

## Methods

### EMF device

**Fig. 1:**
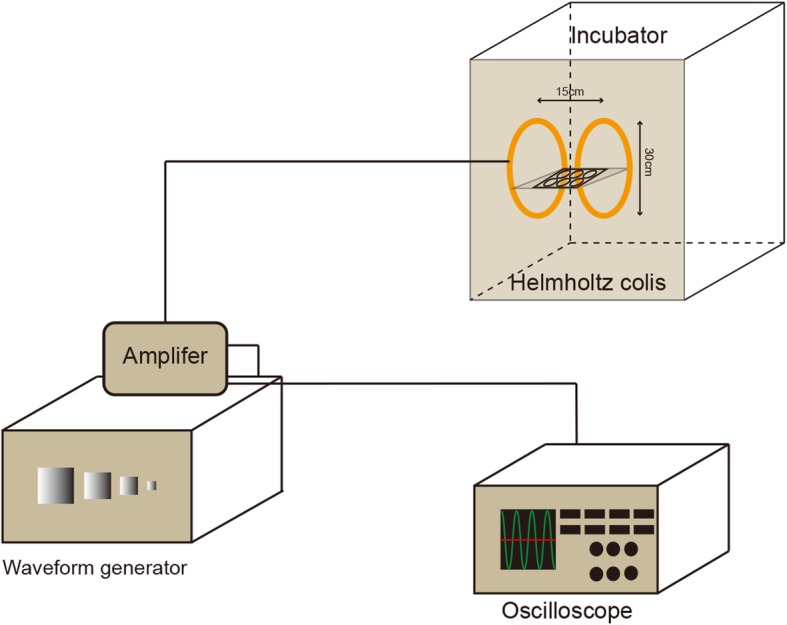
Presentation of the device used to generate the electromagnetic fields (EMFs). The device was constituted of waveform generator, amplifier, oscilloscope, and Helmholtz coils. Cells were placed in the center of the Helmholtz coils, which were placed in a 5% CO_2_ and 37 °C incubator, where the intensity of SEMF is uniform (90%). The temperature is nearly the same as other incubators (within 0.2–0.8 °C)

The sinusoidal EMF (SEMF) facility was designed and manufactured by the Naval Engineering University of China (Fig. 1, Additional file 1). The device was constituted of waveform generator, amplifier, oscilloscope, and Helmholtz coils. Signals created by the waveform generator, after being amplified, were transferred to the coils. The Helmholtz coils which produced EMF were placed in a CO_2_ incubator. Culture plates were placed in the center of coils. A continuous sinusoidal EMF with the constant parameters (1 mT, 15 Hz, 4 h/day) was used in the study [29]. The shame exposed control samples were kept in another incubator with the same conditions without using EMF. The temperature differences inside the two incubators were within 0.2–0.8 °C.

### rBMSC isolation, characterization, and stimulation

Four-week-old (male, 70–100 g) Sprague-Dawley rats were obtained from the Laboratory Animal Center of Tongji Hospital of Hubei Province in China. All experimental processes were performed following international guidelines for the care and use of laboratory animals and were approved by the Ethics Committee of Huazhong Science and Technology University. The rBMSCs were isolated from the femurs and tibiae according to the procedure as described previously [27]. Cells were then resuspended and cultured in Dulbecco’s modified Eagle’s medium F12 (DMEM/F12; Gibco, USA) supplemented with 10% fetal bovine serum (FBS; Gibco) and 1% (v/v) penicillin/streptomycin (P/S; Sigma-Aldrich, USA) at 37 °C in a humidified 5% CO_2_ incubator. The medium was completely changed every 3 days. When reached approximately 80% confluence, cells were passaged and used for the following experiments from second to third passages.

For tri-differentiation analysis, rBMSCs were cultured with inductive medium respectively and evaluated by histological staining after 3 weeks differentiation according to the previous study [30]. The results are shown in Additional file 2.

To investigate the effect of SEMF on osteogenic differentiation of rBMSCs, cells were seeded in sterile six-well plates at an initial density of 5 × 10^4^ cells/well and cultured in DMEM/F12 with 10% FBS, 0.1 μM dexamethasone, and 10 mM β-glycerophosphate (Sigma-Aldrich, USA). Then, cells were exposed to 15 Hz, 1 mT sinusoidal EMF 4 h per day for a week. To investigate the effect of VEGF on angiogenic differentiation of rBMSCs, cells were cultured in DMEM/F12 supplemented with 10% fetal bovine serum, 1% penicillin/streptomycin, and 50 ng/ml VEGF for 1 week (R&D system, American) [31, 32].

### Cell proliferation assay

Cells were seeded at 5 × 10^3^ cells/cm^2^ in 24-well plates. After inducted by VEGF and SEMF or not for 1, 4, and 7 days, the relative cell number was analyzed by the Cell Counting Kit-8(CCK-8; Boster, China) according to the standard protocol. Briefly, 10 μl CCK-8 solution with 100 μl culture medium was added into each well. After 2 h incubation, optical density (OD) absorbance was read at 450 nm by microplate reader (Bio-Rad, USA).

### Alkaline phosphatase (ALP) activity assay

Cells were cultured in 6-well plates at a density of 10,000 rBMSCs per well. After inducted for 1, 4, and 7 days, samples were rinsed twice with PBS, and then, each sample was treated with 1 ml 1% Triton X-100 and shaken slowly on a shaker for 30 min. ALP activity assay was measured according to the manufacturer’s instructions of ALP assay kit (Jiancheng Technology, Nanjing, China). Briefly, 30 μl double distilled water, 0.1 mg/ml phenol standard application solution, and samples were respectively added to the blank wells, standard wells, and sample wells of the 96-well plate. Then, 50 μl buffer and 50 μl substrate solution were added per well. After bathed at 37 °C for 15 min, 150 μl color reagents was finally added to each well and the optical density (OD) value was measured by microplate reader at 520 nm. The protein concentration was measured according to the instruction of bicinchoninic acid (BCA) protein concentration assay kit (Boster, China). The relative ALP activity was obtained by normalizing the ALP amounts to the corresponding total protein contents.

### Western blot analysis

After 1 week induction, cells were rinsed with pre-cooled PBS and lysed using radioimmunoprecipitation assay lysis buffer (RIPA, Boster, China) containing 1% phosphatase and protease inhibitors. The protein concentration of the lysate was determined using a BCA protein assay kit. Cell lysates (20 μg protein) were resolved onto SDS-polyacrylamide gels (8–10%) and transferred onto 0.45-μm polyvinylidene difluoride (PVDF) membranes (Millipore, USA). After blocked with 5% bovine serum albumin (BSA) in Tris-buffered Saline-Tween solution (TBST) for 1 h at room temperature, the blots were incubated with the specific primary antibodies (OPN, RUNX2, COLI, VEGFR2, vWF, CD31, WNT1, and LRP-6 at 1:1000 dilution, β-catenin at 1:2000 dilution, Abcam, UK; GAPDH at 1:500 dilution, Boster, China) at 4 °C overnight. The membranes were next incubated with the corresponding horseradish peroxidase-conjugated goat anti-rabbit (1:5000 dilution) or goat anti-mouse (1:5000 dilution) antibodies (Boster, China) at 37 °C for 1 h after rinsing with TBST. The blots were then performed with an enhanced chemiluminescence (ECL, Thermo Fisher Scientific, USA). Relative expression was quantified using the Image Lab system version 5.1 (Bio-Rad Laboratories, USA) and normalized to GAPDH.

### Quantitative real-time PCR (Q-PCR)

Q-PCR was used to evaluate the gene expression of inducted cells. Total RNA was extracted with the EZNA Total RNA kit (Omega Bio-Tek, USA), and single-stranded cDNA was synthesized by Reverse Transcription kit (Toyobo Life Science, Japan). Expression of VEGFR2, VWF, CD31, OCN, COLI, and Runx2 were quantified using a CFX96 (Bio-Rad Laboratories, USA) and the SYBR Green Real-Time PCR Master Mix (Toyobo Life Science, Japan). Target genes were normalized to corresponding GAPDH levels, and the cDNA samples were triplicates. The sequences of primers are listed in Table 1.

**Table 1:** List of primer sequences used in this study

Gene	Forward(5′–3′)	Reverse(5′–3′)
VEGFR2	CACGGGAAACTACACCGTCA	TCCACAGGCGAGATCAGAGA
vWF	TCTTCCAGGACTGCAACAAG	TCCGAGATGTCCTCCACATA
CD31	CACAGCAATTCCTCAGGCTA	TTCAGCCTTCAGCATGGTAG
OCN	GGAGGGCAGTAAGGTGGTGA	GAAGCCAATGTGGTCCGC
RUNX2	CTACTCTGCCGAGCTACGAAAT	TCTGTCTGTGCCTTCTTGGTTC
COLI	CTTCTGGCCCTGCTGGAAAGGATG	CCCGGATACAGGTTTCGCCAGTAG
GAPDH	CCGCCCAGAACATCATCCCT	GCACTGTTGAAGTCGCAGGAGA

### Immunofluorescence

Bone marrow mesenchymal stem cells were seeded in six-well plate at 2 × 10^4^ cells per well. After 7 days induction, cells were fixed with 4% paraformaldehyde for 15 min, treated with 0.1% Triton X-100 for 20 min, and blocked 5% BSA for 35 min at 37 °C. Cells were then washed three times with PBS and incubated with CD31 (R&D systems, USA, 1:200 dilution) and OCN (R&D systems, USA, 1:100 dilution) at 4 °C overnight. After rinsing, cells were incubated with FITC-conjugated rabbit anti-goat secondary antibody (Boster, China, 1:50 dilution) and CY3-conjugated goat anti-mouse secondary antibody (Boster, China, 1:50 dilution) in dark for 1 h at 37 °C. Finally, after washed three times with PBS, cells were stained with 4–6-diamidino-2-phenylindole (DAPI) for 10 min, then rinsed with PBS twice and analyzed under a fluorescence microscope (EVOS FL Auto, Life Technologies, USA).

### Fabrication of composite PCL/HA scaffold

A composite consisting of PCL (Mn 80,000 pellet, Sigma, USA) and HA (less than 200 nm size particles, Sigma, USA) with weight ratio of 3:2 was dissolved in the chloroform. The mixture was agitated by magnetic stirrer at 400 rpm for 5 h at room temperature (26 ± 1 °C). Then, the paste mixture was fabricated using an air pressure 3D bio-printer (Fochif Tech, China). Cylindrical scaffolds (4 mm diameter, 1 mm thick) with 3D orthogonal periodic porous architectures were designed using Mimics Software (Materialise, BE). The fiber diameter and spacing between fibers was 200 μm and 300 μm, respectively, and the layer thickness was 200 μm. The layer deposition angle was modified from 0° to 90° after one layer.

### Cell morphology on PCL/HA scaffold

For observing cell morphology, a suspension of 2 × 10^5^ cells in 200 μl medium was loaded onto PCL/HA scaffold in 24-well plates before induction. After 3 days induction, the scaffolds were fixed with glutaraldehyde solution for 15 min. Following the removal of the glutaraldehyde solution, scaffolds were rinsed three times with PBS. Then, the samples were dehydrated with increasing concentration of ethanol solution for 15 min at each procedure and dipped in isoamyl acetate for 20 min. Then, samples were critical-point dried, mounted on specimen stubs, and coated with gold prior to scanning electron microscopy (SEM, U8010, HITACHI) examination.

### In vivo calvarial bone defect model

Sixty-eight Sprague-Dawley (SD) rats weighing 150–180 g bought from the Experimental Animal Center of Huazhong University of Science and Technology (Wuhan, Hubei, China) were applied. After anesthetization by intraperitoneal injecting of pentobarbital at a concentration of 3.5 mg/100 g, a 0.8–1.6-cm sagittal incision was made on the scalp and two 4 mm subcritical defect was created with a trephine bur [33]. Defects were approximately 2 to 3 mm apart from each other. Then, the sterile constructs with or without cells were implanted into the cranial defects, and the uniform bone defects were randomly allocated into six groups: (1) blank defect (*n* = 8); (2) acellular PCL/HA scaffold, PCL/HA (*n* = 12); (3) PCL/HA scaffold seeded rBMSCs, PCL/HA/MSCs (*n* = 12); (4) PCL/HA scaffold seeded rBMSCs with 50 ng/ml VEGF induction in vitro, PCL/HA/MSCs/VEGF (*n* = 12); (5) PCL/HA scaffold seeded rBMSCs with SEMF(1 mT, 15 Hz, 4 h/day) induction in vitro, PCL/HA/MSCs/SEMF (*n* = 12); and (6) PCL/HA scaffold seeded rBMSCs with SEMF and VEGF induction, PCL/HA/MSCs/SEMF/VEGF (*n* = 12). The incision was sutured in layers after operation. All cell-loaded constructs were seeded cells at the same time. Afterwards, the intervention groups were inducted for 1 week before implantation. Control group was also incubated for 1 week before implantation in the same condition. All surgeries were finished in 3 days.

### Microcomputed tomography (micro-CT) scanning

Four rats for each group at each time point were euthanized at 4 and 12 weeks. The harvested specimens were scanned with micro-CT. Micro-CT scanning was performed using the following conditions: 70 mA, 120 kV, and 15 μm. The images were reconstructed using the built-in software. The bone mineral density (BMD) and bone volume/total volume (BV/TV) within the defect regions were analyzed (*n* = 4).

### Histological evaluation

Four and 12 weeks after operation, four rats of each group at each time point were sacrificed and the cranial samples were dipped in 4% neutral paraformaldehyde solution for 2 days. After decalcifying and embedding, specimens were sliced with a thickness of 5 μm. The slices were stained with hematoxylin and eosin (HE) and Masson’s trichrome (Masson) to estimate new bone and vessel formation.

### Mechanical push-out testing

To ascertain the interfacial shear strength of the porous scaffolds, a push-out test was performed on an Instron 5566 device (Instron Corporation, USA) after 12 weeks surgery. Four samples from each implanted constructs groups were harvested for the push-out test. The crania of four normal 20-week-old SD rats were introduced as the normal control. Each specimen was fixed on a custom-made support jig with a 5-mm hole to minimize the effect of the testing jig on the results. A vertical force was applied on the constructs at a constant displacement speed of 0.5 mm/min until the implants loosed. Ultimate force (F) and load-displacement curve were obtained. Ultimate stress (σ) was calculated according to the previously reported formula [34].

### Statistical analysis

All data are presented as mean ± standard deviation. SPSS version 19.0 (IMB Corp., USA) was used to analyze the statistical significance between different groups. Data from each time point were analyzed by one-way analysis of variance (ANOVA) and Bonferroni’s post hoc test. *p* < 0.05 was considered as statistically significant.

## Results

### Cell morphology

**Fig. 2:**
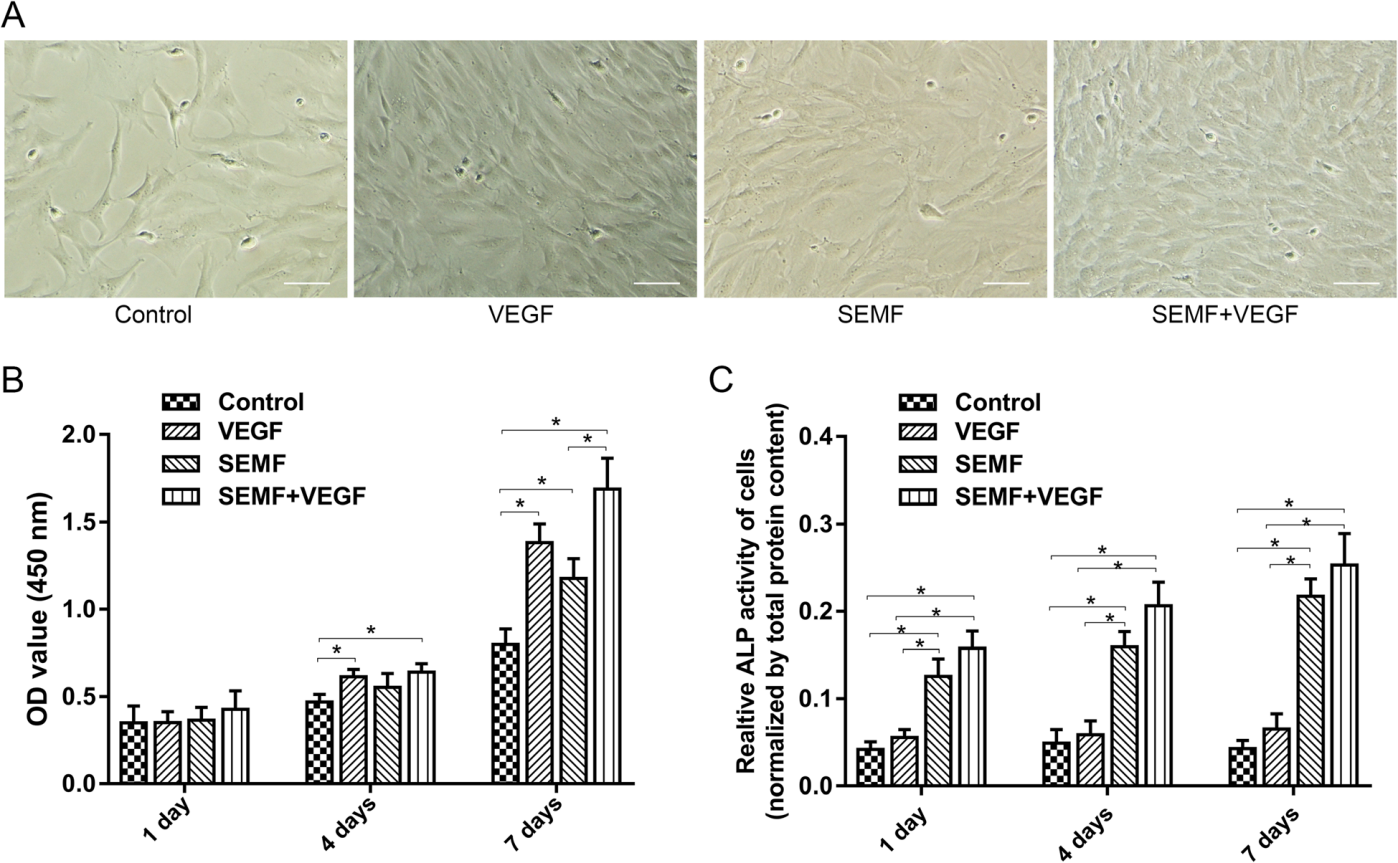
Morphology of rBMSCs observed under microscope after 1 week induction of SEMF (1 mT, 15 Hz, 4 h/day) and VEGF (50 ng/ml). Scale bar = 100 μm (**a**). Proliferation of MSCs treated with SEMF and VEGF was tested using CCK-8 at 1, 4, and 7 days (**b**). Relative ALP activity of rBMSCs inducted by SEMF and VEGF at 1, 4, and 7 days (**c**). Data represents mean ± SD (*n* = 3, *p < 0.05)

After 1 week induction with or without SEMF or VEGF, third generation rBMSCs showed some morphological changes between different groups. rBMSCs showed a typical shape of spindle in control and SEMF-inducted groups, while cells in VEGF-inducted group revealed a short spindle or pebble morphology and in SEMF/VEGF-inducted group observed a cobble-like morphology (Fig. 2a).

### Effects of VEGF and SEMF on proliferation of rBMSCs

To explore the effects of VEGF and SEMF on proliferation of rBMSCs, CCK-8 assay was used. The results showed that rBMSCs proliferated increasingly with time in four groups. No significant difference about cell numbers was observed between four groups after 1 day incubation. Compared with the control group, VEGF alone and VEGF associated with SEMF groups exhibited an increased proliferation, while SEMF group showed no significant difference after 4 days induction. With 1 week induction, all inducted groups showed increasing cell numbers in comparison to the control group and the SEMF-associated VEGF group showed a higher proliferation level in contrast to the SEMF group (Fig. 2b).

### Effects of VEGF and SEMF on rBMSC ALP activity

To investigate the effects of VEGF and SEMF on rBMSCs in vitro osteogenesis, ALP activity was evaluated by ALP assay kit. The SEMF alone and SEMF/VEGF groups demonstrated a significant increase in ALP activity expression compared with the control and VEGF groups at each time point, and the expression of ALP activity increased gradually with time in these two groups. However, no significant differences between the SEMF group and SEMF/VEGF group were shown (Fig. 2c).

### VEGF and SEMF promote the expression of osteogenesis- and endothelia-related markers at the protein and gene level

**Fig. 3:**
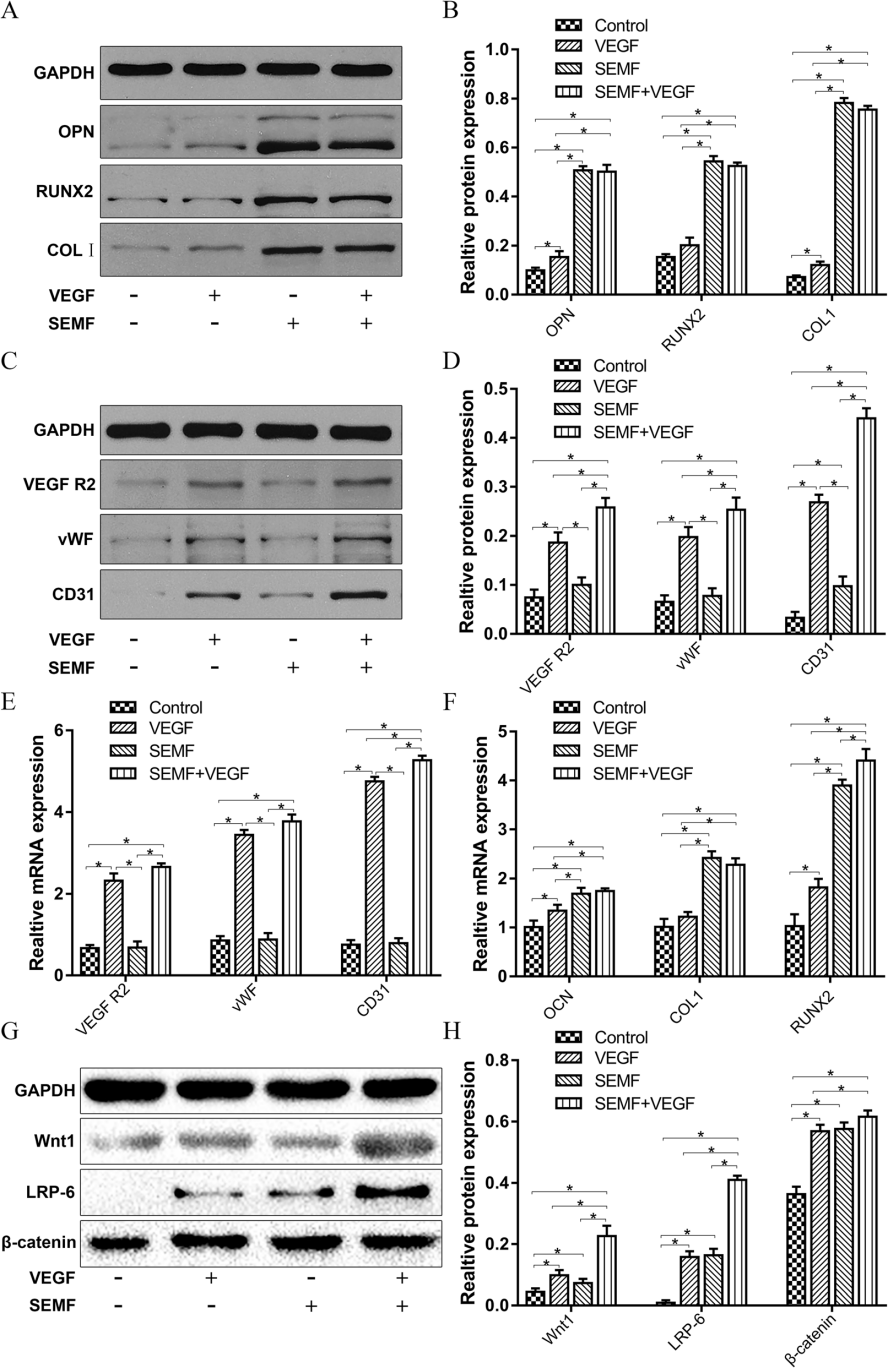
The amounts of OPN, RUNX2, COLI, VEGFR2, vWF, and CD31 were evaluated with western blot after 1 week induction of EMF and VEGF (**a**, **c**). The expression ratios of those proteins were quantitatively analyzed (**b**, **d**). The mRNA expression levels of VEGFR2, vWF, CD31, OCN, RUNX2, and COLI were detected using Q-PCR after 1 week treatment of SEMF and VEGF (**e**, **f**). The expression of Wnt1, LRP-6, and β-catenin were evaluated through western blotting (**g**). The protein expression ratios were quantitatively analyzed (**h**). Data are shown as mean ± SD (*n* = 4, *p < 0.05)

After 1 week culture and induction, synthesis of osteogenesis- (OPN, RUNX2, COLI) and endothelia-related (VEGFR2, vWF, CD31) proteins was analyzed by western blot analysis. The lowest level of OPN, RUNX2, and COLI was observed in the control group. The expression levels of those proteins in the presence of SEMF exposure group increased significantly compared to that without SEMF exposure (*p* < 0.05, Fig. 3a, b). The SEMF group showed no significant difference with the SEMF/VEGF group in those protein expressions. Notably, the VEGF group showed a higher expression of OPN and COLI in comparison with the control group. Endothelia-related proteins increased significantly in the VEGF-inducted group. Expression of proteins in the SEMF/VEGF group exhibited the highest level. Also, in the SEMF group, the CD31 expression enhanced significantly in contrast to the control group (Fig. 3c, d).

For the regulation of osteogenesis- (OCN, RUNX2, COLI) and endothelia-related (VEGFR2, vWF, CD31) genes, cell lysates were detected by Q-PCR. It was demonstrated that cell cultured with SEMF induction exhibited higher osteogenesis genes than other two groups. With SEMF and VEGF induction, cells showed a significant increase in RUNX2 expression, while no significant difference in OCN and COLI expression, compared with only SEMF induction group. Notably, cell cultured with VEGF induction also expressed higher RUNX2 and OCN than the control group (*p* < 0.05, Fig. 3f). For endothelia gene expression, the VEGF-inducted groups increased significantly in contrast with other two groups. In comparison with the VEGF group, the SEMF/VEGF group exhibited higher expression in CD31 while no significant difference in VEGFR2 and vWF (*p* < 0.05, Fig. 3e).

**Fig. 4:**
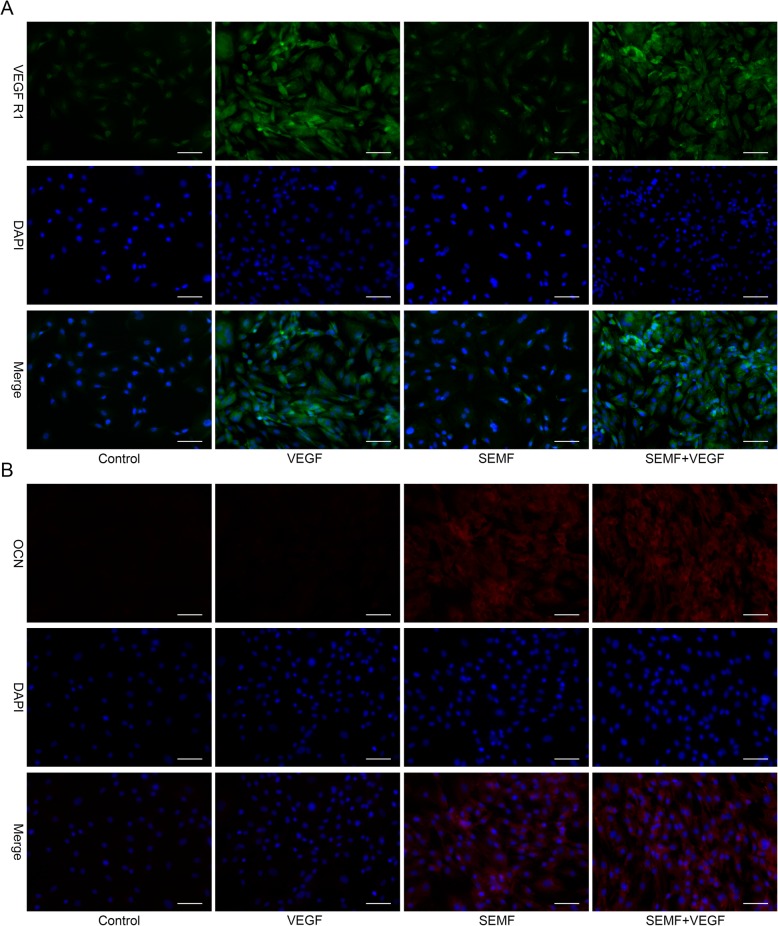
The expression of VEGFR1 (green) and OCN (red) in rBMSCs after 7 days stimulation of VEGF and SEMF were observed by immunofluorescence staining (**a**, **b**). The nuclei were stained with DAPI (blue). Scale bar = 100 μm

The expression levels of OCN and VEGFR1 were then investigated by immunofluorescence. Concordant with gene and protein expression data, OCN and VEGFR1 deposition in cells were markedly increased by exposure to SEMF and VEGF, respectively (Fig. 4a, b).

### Wnt pathway is involved in osteogenic and angiogenic activity of rBMSCs exposed to SEMF and VEGF

As shown in Fig. 3g, h, the expression of Wnt1, LRP-6, and β-catenin increased significantly in inducted groups. The SEMF/VEGF group exhibited the highest level of these proteins in all groups, while showing no significant difference in β-catenin compared with the SEMF group. The synthesis of these proteins demonstrated no significant difference in the VEGF and SEMF group.

### Cell morphology on PCL/HA scaffold

**Fig. 5:**
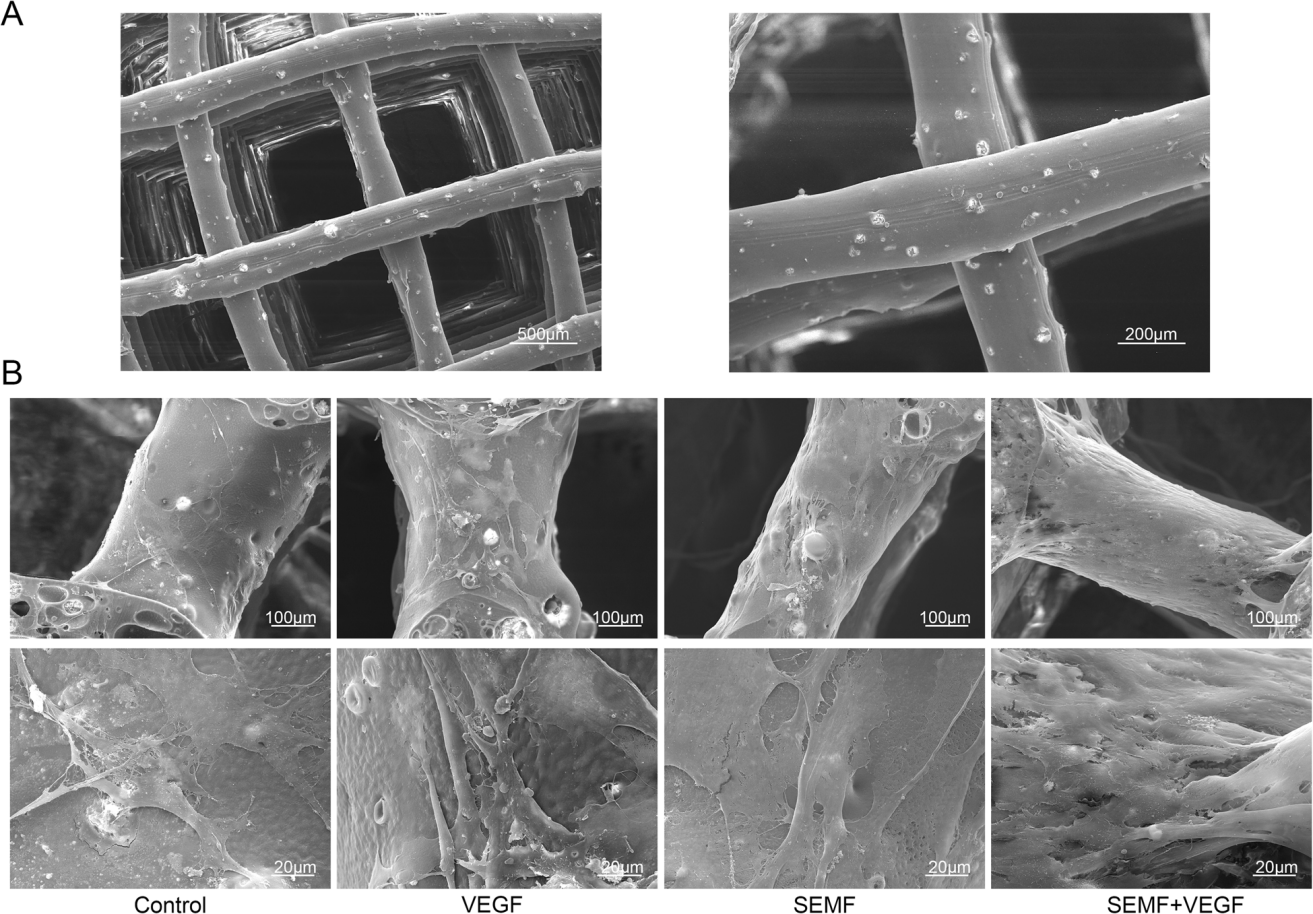
SEM morphology of the polycaprolactone/hydroxyapatite scaffold (**a**). Scale bar, 500 μm and 200 μm. SEM images of cell morphology on PCL/HA scaffold after 3 days of SEMF and VEGF induction (**b**). Scale bar, 100 μm and 20 μm

SEM images showed that PCL/HA scaffold had a square pore structure and fibers from upper and lower layers arranged vertically (Fig. 5a). After 3 days induction, cell morphologies on PCL/HA scaffold are observed in Fig. 5b. Cells adhered to the PCL/HA fibers and extended pseudopodia. Cell proliferation in the SEMF/VEGF group increased observably compared with other groups. And there were no significant differences in cell morphology between all groups.

### Micro-CT evaluation findings

**Fig. 6:**
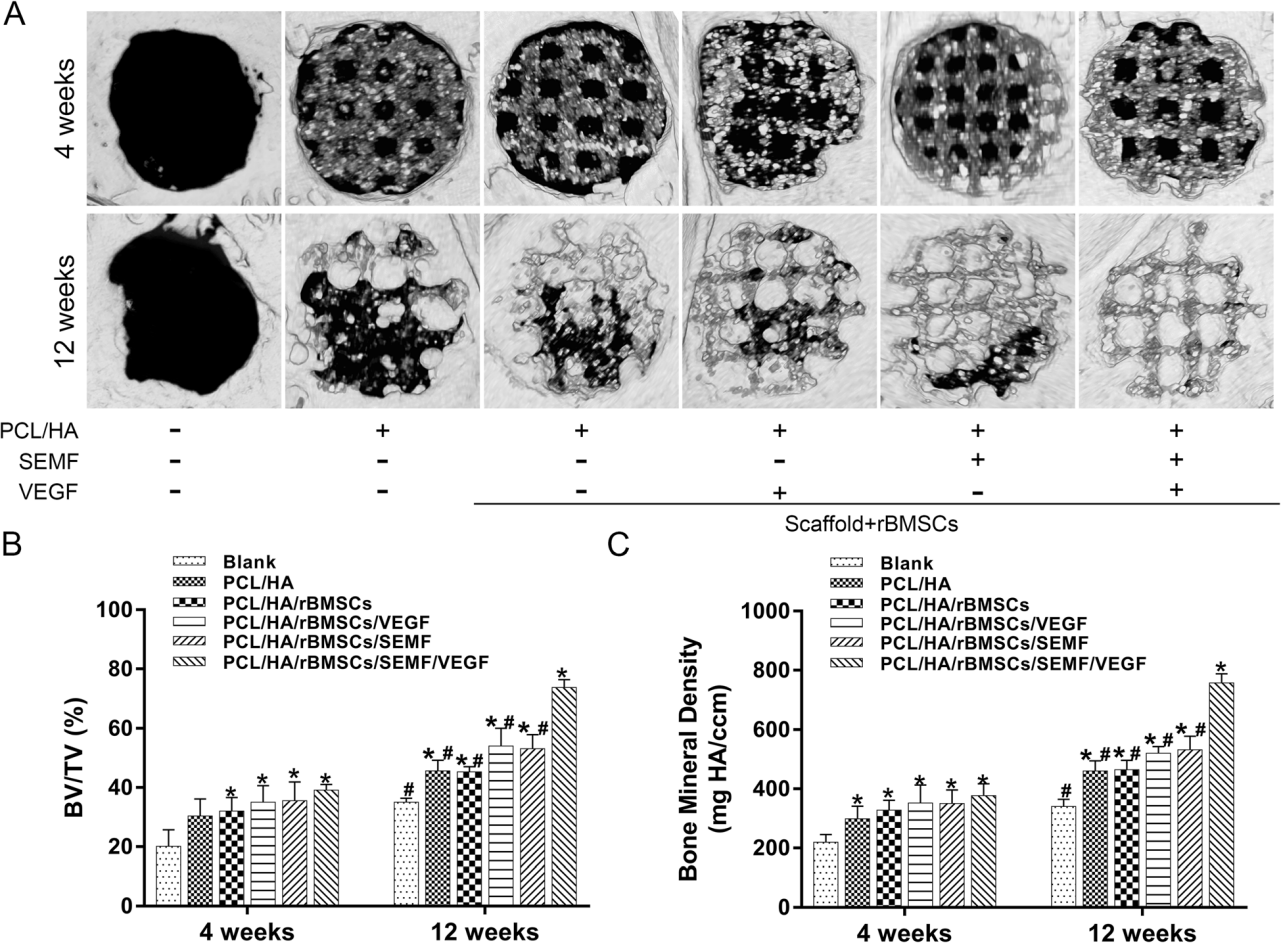
Three-dimensional reconstruction images (**a**) of the defect sites by micro-CT in different groups at 4 and 12 weeks. BV percentage (**b**) and BMD in the implanted scaffolds (**c**) were quantified (*p  < 0.05 compared with the control group, #*p*  < 0.05 compared with the co-inducted group)

The reconstructed 3D images showed that limited bone formation was observed in the blank group at 4 and 12 weeks (Fig. 6a). At 4 weeks after operation, only a few new bones were formed in three inducted implanted group at the edge of defect and there is no significant difference between those groups. Obvious bone remodeling and osteointegration occurred at 12 weeks after implantation, and evident bone formation was observed in the PCL/HA/rBMSCs/SEMF/VEGF group in comparison with other groups (Fig. 6a). The morphometrical analysis showed that significantly greater BMD and BV/TV were detected for the five implanted groups than the blank group at 4 weeks (Fig. 6b, c). BMD and BV/TV increased significantly in SEMF/VEGF group at 12 weeks in comparison with other implanted groups. No significant difference was observed in PCL/HA, PCL/HA/rBMSCs, PCL/HA/rBMSCs/VEGF, and PCL/HA/rBMSCs/SEMF.

### Histological evaluation

**Fig. 7:**
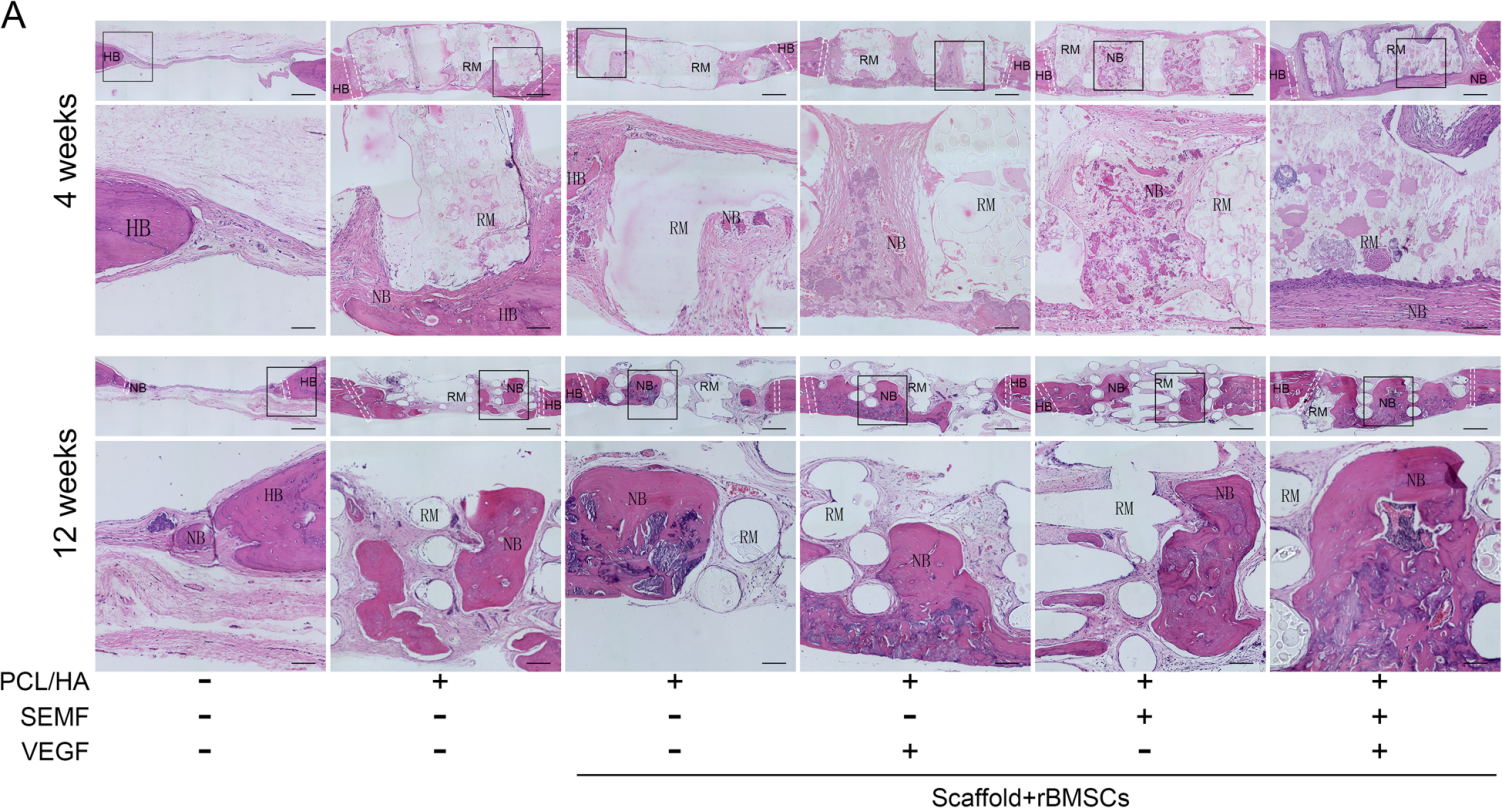
H&E staining of rat cranial defect repair tissue sections in the defect region was taken 4 and 12 weeks post-operation to evaluate new bone ingrowth and intimate contact with host bone. HB, host bone; NB, new bone; RM, residual material. The dotted rectangles designate the interface of the implanted constructs with host tissue. The black boxes represent the regions of enlargement. Scale bar = 200 μm

**Fig. 8:**
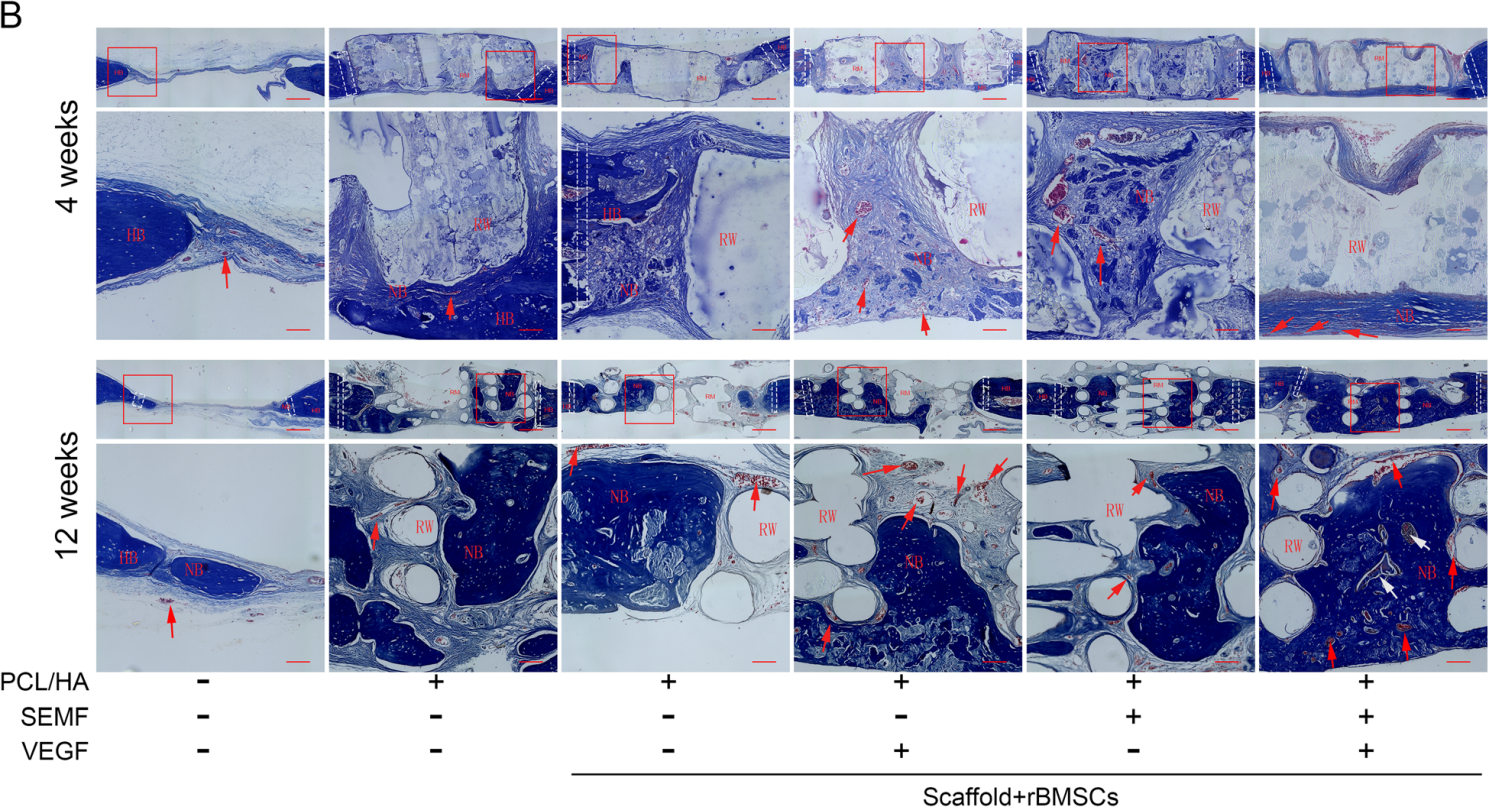
Masson’s staining of rat cranial defect 4 and 12 weeks post-operation. HB, host bone; NB, new bone; RM, residual material. The dotted rectangles designate the interface of the implanted constructs with host tissue. The red boxes represent the regions of enlargement. The red arrows represent the newborn blood vessels in collagen fibers. The white arrows indicate that the bone marrow cavity was grown into the new bone. Scale bar = 200 μm

The histological characteristics are observed through H&E and Masson’s staining in all groups in Figs. 7 and 8. The staining revealed that plenty of scaffold materials and connective tissue could be observed in implantation groups at 4 weeks post-implantation. Newly formed bone was evidently observed in the PCL/HA/rBMSCs/SEMF/VEGF group, whereas only little new bone formation was observed in peripheral regions of the defects in other implanted groups (Fig. 7). After 12 weeks, degraded scaffolds remnants, connective tissue, and regenerate bone could be seen in all implantation groups. Also blood vessels of medium-large size could be observed in the implanted groups, especially in the PCL/HA/rBMSCs/VEGF and PCL/HA/rBMSCs/SEMF/VEGF groups, in Masson’s staining (Fig. 8). Extensive area of regenerate bone and mineralized bone trabeculae of normal morphology was obviously observed in the PCL/HA/rBMSCs/SEMF/VEGF groups, which indicated a high bone forming activity. Notably, continuous bone was formed between implanted constructs and host cortical bone. However, uncontinuous large osteoid islands with partial scaffolds were observed in the PCL/HA/rBMSCs/SEMF and PCL/HA/rBMSCs/VEGF groups. No obvious difference in new bone formation was observed in the two groups. Smaller osteoid islands and larger scaffolds could be seen in the PCL/HA/rBMSC group compared with the intervention group. A plenty of scaffold remnants were observed in the PCL/HA group, and partially new bone was noticed (Figs. 7 and 8).

### Mechanical push-out testing

**Fig. 9:**
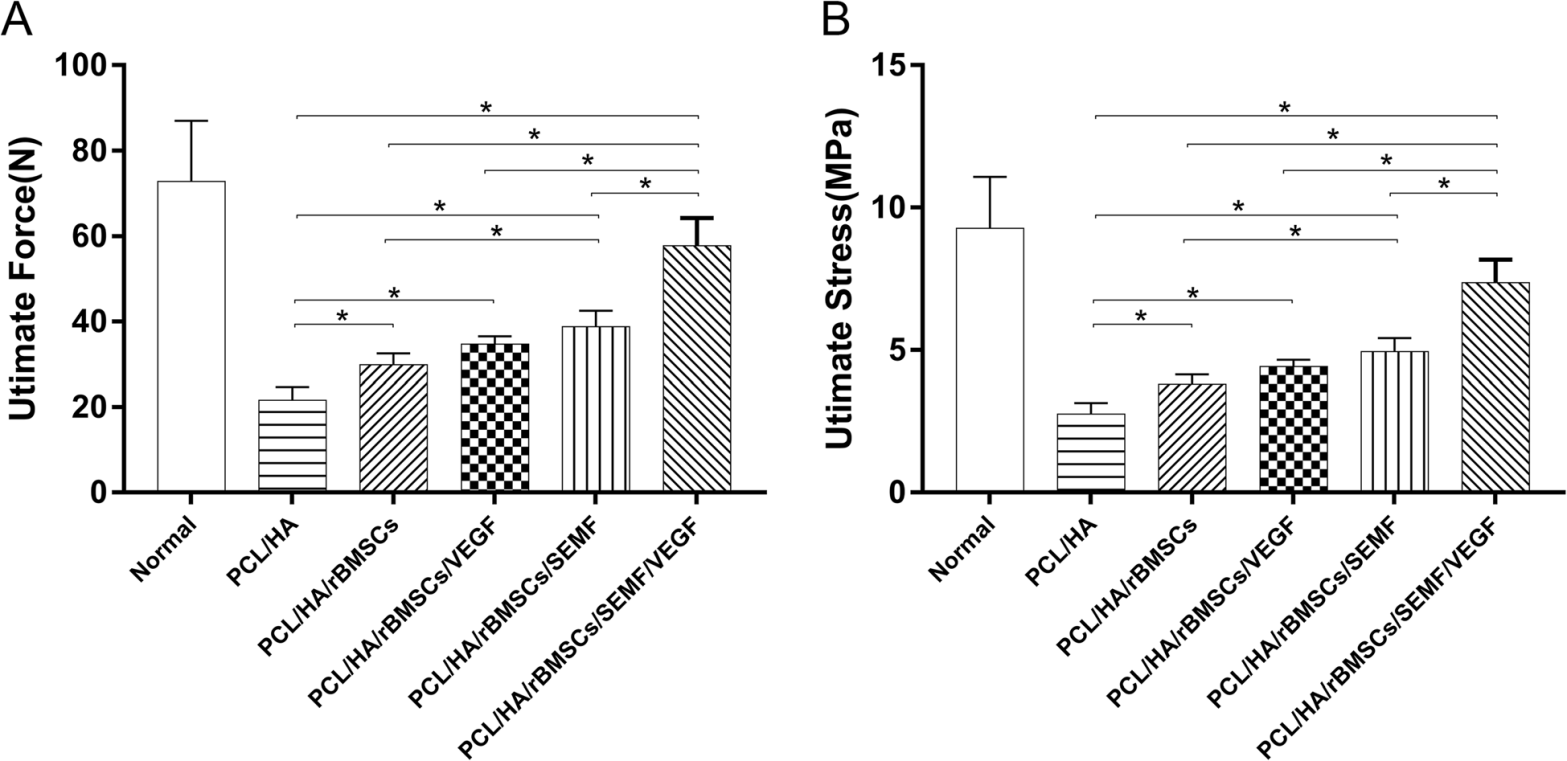
Twelve weeks after surgery, the biomechanical properties of the cranium were performed by push-out testing. Immediately after harvest, constructs were placed on a custom testing jig and a vertical force was applied. The ultimate force (**a**) and ultimate stress (**b**) required to displace the implants from the surrounding tissues were recorded. Data are shown as mean ± SD (*n* = 4, *p < 0.05)

The biomechanical properties of the crania were tested by push-out testing 12 weeks post-implantation. The results showed that the PCL/HA/rBMSCs/SEMF/VEGF group had significantly higher ultimate force and ultimate stress compared to other implantation groups (*p* < 0.05; Fig. 9a, b). Besides, the ultimate force and stress in revealed the PCL/HA/rBMSCs/SEMF and PCL/HA/rBMSCs/VEGF group significantly greater than the PCL/HA/rBMSCs and PCL/HA group. And there is no significant difference between the two groups.

## Discussion

Tissue-engineered bone, consisted of stem cells, scaffolds, and appropriate induction, is considered a potential strategy for bone defect restoration and is widely investigated recent decades. However, insufficient vascularization and slow bone regeneration restrict the development of tissue-engineered bone in clinical application. To tackle the two challenges, a plenty of studies focused on the isochronous effect of osteogenesis and vascularization has been investigated recent years [35–37]. It is reported that dimethyloxaloylglycine (DMOG)-loaded mesoporous silica nanospheres (MSNs) could promote the osteogenesis and angiogenesis of human bone marrow stromal cells (hBMSCs) by Si ions and DMOG [35]. Furthermore, synergistic effect of BMP-2 and VEGF enhanced the mineralization and vascularization in hydrogel and regeneration of vascularized bone [37]. In this study, our findings demonstrated that the application of SEMF and VEGF could enhance the expression of osteogenic- and endothelial-related markers of rBMSCs by activating Wnt/β-catenin pathway in vitro, respectively. Further, the fabricated inducted rBMSC-laden PCL/HA constructs were employed to repair rat subcritical cranial defects in vivo*.* According to the micro-CT evaluations and histological studies results, enhanced bone regeneration and vascularization in the implanted PCL/HA/rBMSCs/SEMF/VEGF constructs were confirmed.

As multipotent stem cells, BMSCs are ideal cell resources for tissue engineering. In many previous studies, BMSCs seeded scaffolds with different stimuli could promote the bone regeneration and vascularization of bony defect region, such as BMP-2, VEGF, HIF-1α, and Si [35, 37, 38]. The therapeutic effects of EMF in enhancing bone regeneration have been widely investigated and acknowledged for decades. To achieve a better bone defect recovery, tissue materials combined with EMF have also been investigated recent years. However, the overwhelming majority of studies focused on the pulsed electromagnetic fields (PEMF) with biomaterials to enhance osteogenesis [39]. Although sinusoidal electromagnetic fields (SEMF) also could increase the osteogenic differentiation of rBMSCs [27–29], the combined effect of SEMF with bioscaffolds to restore the bone defect has not yet been reported. In the present study, SEMF associated with VEGF was introduced to promote the vascularization of tissue-engineered bone.

The results in Figs. 2c and 3a–f demonstrated that the induction effect of SEMF and VEGF enhanced the osteogenic- and vasculogenic-related protein and gene expression of rBMSCs, respectively. The osteogenic-related proteins and genes showed a significant increase in groups with SEMF exposure. Our observations was in coincidence with the results of Ledda et al.’s study, which reported that the nonpulsed sinusoidal EMF exposure was able to induce the osteogenic differentiation of human MSCs [26]. However, the gene expression of RUNX2 increased in the SEMF/VEGF group compared with the SEMF group. Moreover, some protein (OPN, COLI) and gene (OCN, RUNX2) expression increased in the VEGF group compared to the control group. These results indicated that VEGF might have slight effect on expression of some osteogenic-related proteins and genes. This phenomenon was similar with some previous study, which reported that VEGF-C could induce the osteogenic differentiation of human mesenchymal stem cells through the ERK and RUNX2 pathway [40]. The angiogenic-related protein and gene expression also enhanced significantly in the VEGF-inducted groups. The expression of angiogenic proteins increased significantly in the SEMF/VEGF group compared with the VEGF group. However, only CD31 gene expression showed the same trend. In our study, we did not find that SEMF could promote the angiogenic differentiation of rBMSCs alone. Additionally, no obvious synergetic effect was observed between EMF and VEGF in angiogenic differentiation. They just slightly promoted angiogenic differentiation according to the results about related gene and protein expressions. This effect may also associate with RUNX2 functions, as RUNX2 overexpression in mesenchymal cells increased VEGF expression under both normoxic and hypoxic conditions [41].

It is widely investigated that Wnt/β-catenin pathway participated in mediation osteogenic differentiation of MSCs [42, 43]. It was reported that SEMF could enhance osteoblast differentiation of MSCs through Wnt/β-catenin pathway [27, 44]. Wnt/β-catenin pathway also was involved in vasculogenesis and angiogenesis [45]. Moreover, VEGF mediated the angiogenic induction of MSCs through Wnt signal pathway [16, 46, 47]. Therefore, the expression of Wnt signal pathway-related markers Wnt1, LRP-6, and β-catenin were measured in our study (Fig. 3g, h). The protein expression increased significantly in the inducted groups compared with the control group, especially in the SEMF/VEGF group. No significant differences were revealed in the protein expressions in SEMF group and VEGF group. Those data suggest that both SEMF and VEGF promote the differentiation of MSCs through Wnt/β-catenin pathway.

As PCL is a non-toxic polyester and has the properties of biodegradability and plasticity, it is extensively used in tissue engineering [48]. Moreover, PCL/HA scaffold significantly improved bone regeneration [33]. Composite materials combined with 3D printing technique make it possible to obtain an ideal scaffold with designed structure, modulated mechanical properties of the biomaterial matrix, and optimized performances of biodegradability and bioactivity [49]. Therefore, we used 3D printed PCL/HA scaffold to restore the bone defect in present study.

The data showed that rBMSCs under SEMF and VEGF induction revealed a better proliferation on PCL/HA scaffold compared with other groups (Fig. 5b).The reconstructed 3D images of micro-CT showed a gross overview of implanted constructs with host bones (Fig. 6a). And the quantitative analysis demonstrated that the quantity of BV percentage and BMD was highest in the SEMF- and VEGF-inducted rBMSC-laden PCL/HA group in each time point (Fig. 6b). Histological evaluation of the implantation also revealed that co-inducted MSC-laden PCL/HA group had the greatest bone formation in contrast to other groups (Figs. 7 and 8). The defect restoration area in the SEMF- or VEGF-inducted MSC-laden PCL/HA group was higher compared with the PCL/HA/rBMSCs and PCL/HA groups, but with no significant difference to each other. New blood vessels could be observed in Masson’s staining, especially in 12 weeks. The VEGF-inducted rBMSC-laden PCL/HA group and SEMF combined with VEGF-inducted group showed higher newly formed vessel numbers compared to other groups, no significant difference between the two groups. These results suggested that both SEMF and VEGF pre-inducted scaffolds could promoted the bone formation in bone defect region. Although PCL/HA scaffolds alone could also improve the bone formation of the defect as showed in the present study and the others [33, 50], the restoration of the defect was dissatisfactory. Due to the post-trial effect of SEMF and VEGF on the osteogenic and angiogenic differentiation of rBMSCs, respectively [32, 51], the new bone and vessel formation were increased compared with the group without either stimulus. The possible interpretation for the bone formation enhanced by VEGF was that it enhanced the vessel formation in the defect region, which provided sufficient blood supply for new bone formation. Compared with individual treatment, SEMF/VEGF pre-inducted scaffolds showed a better bone mass and maturity. We considered that SEMF promoted the osteoblast differentiation and VEGF simultaneously enhanced the vasculogenic differentiation. Furthermore, the rapid neovascularization of the engineered constructs greatly improved the bone repair of the defect region finally.

## Conclusions

Through the present study, we demonstrated that SEMF and VEGF induction enhanced the osteoblast differentiation and endothelial differentiation of rBMSCs, respectively. SEMF with VEGF could slightly promote angiogenic differentiation. Moreover, we seeded the rBMSCs onto PCL/HA scaffold. After 1 week induction of SEMF and VEGF, the pre-engineered grafts were implanted into a rat subcritical cranial defect. The enhancement of angiogenesis and bone regeneration was observed. The combinatory effect of SEMF and VEGF raised an efficient approach to enhance the osteogenesis and vascularization of tissue-engineered constructs and provide a useful method to restore bone defects.

## Supplementary information

**Additional file 1.** The photo showed that the device was constituted of waveform generator, amplifier, oscilloscope, and Helmholtz coils. The coils were placed in the incubator. (TIF 6480 kb)

**Additional file 2.** After 3 weeks induction of osteogenic, adipogenic and chondrogenic inductive medium, cells were stained with Alizarin Red S, Oil Red O and Alcian Blue, respectively. Scale bar = 100 μm. (TIF 4893 kb)

## Data Availability

The data used in the current study are available upon request.

## Abbreviations

EMF: Electromagnetic field
VEGF: Vascular endothelial growth factor
rBMSCs: Rat bone marrow-derived mesenchymal stem cells
PCL/HA: Polycaprolactone-hydroxyapatite
LRP-6: Low-density lipoprotein receptor-related protein 6
BMPs: Bone morphogenetic proteins
ALP: Alkaline phosphatase
OPN: Osteopontin
RUNX2: Runt-related transcription factor 2
COLI: Collagen type I
VEGFR2: Vascular endothelial growth factor receptor 2
vWF: von Willebrand factor
CD31: Platelet endothelial cell adhesion molecule-1, PECAM-1
OCN: Osteocalcin
VEGFR1: Vascular endothelial growth factor receptor 1
SEM: Scanning electron microscopy
BMD: Bone mineral density
BV/TV: Bone volume/total volume
