# Supplementary figures and images for: The combinatory effect of sinusoidal electromagnetic field and VEGF promotes osteogenesis and angiogenesis of mesenchymal stem cell-laden PCL/HA implants in a rat subcritical cranial defect
Source: Stem Cell Res Ther. 2019 Dec 16;10:379. doi: 10.1186/s13287-019-1464-x (PMC6915868; doi:10.1186/s13287-019-1464-x)

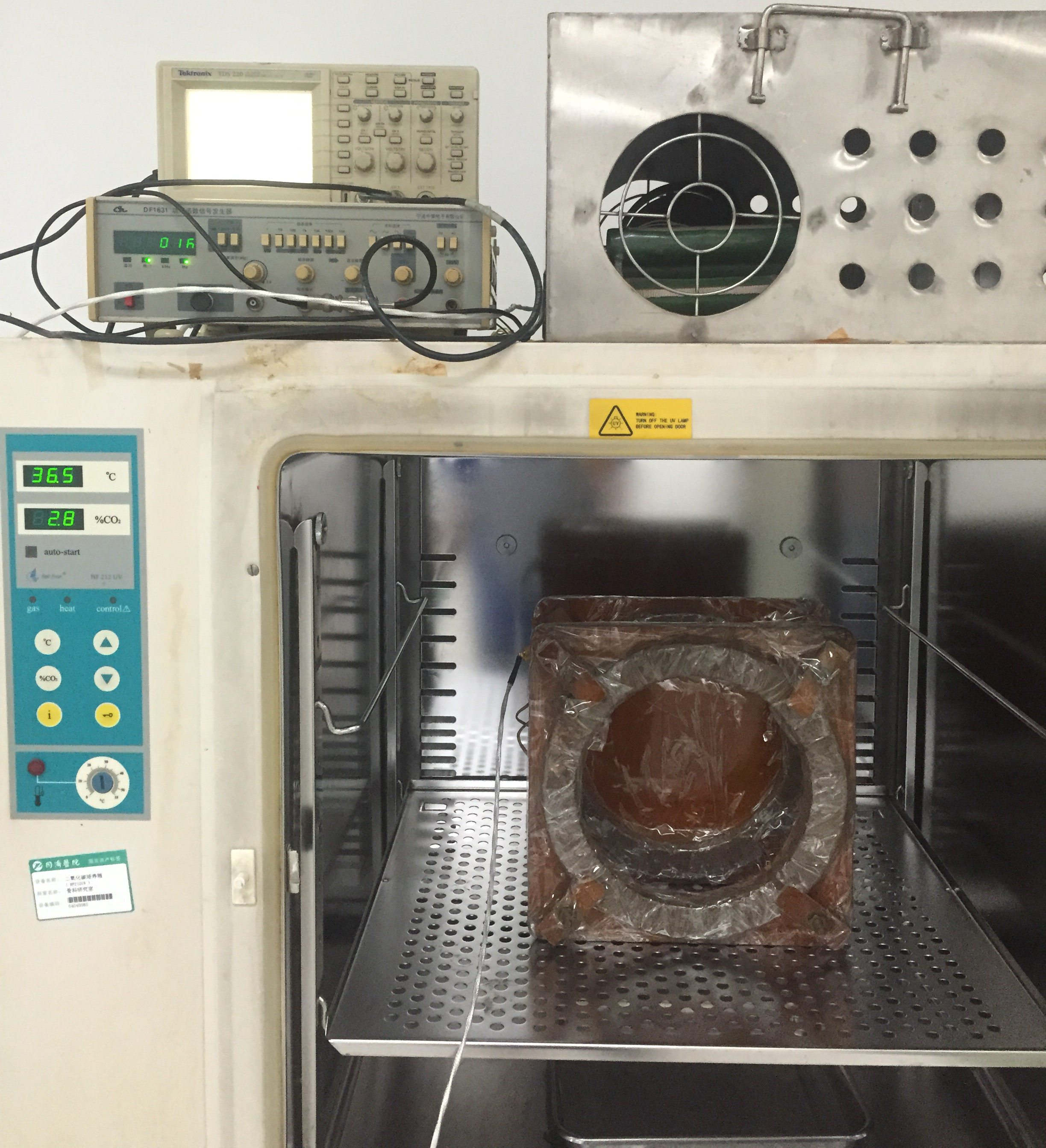

Supplement: Supplementary file 1 — Additional file 1. The photo showed that the device was constituted of waveform generator, amplifier, oscilloscope, and Helmholtz coils. The coils were placed in the incubator. (TIF 6480 kb) [file 13287_2019_1464_MOESM1_ESM.tif]

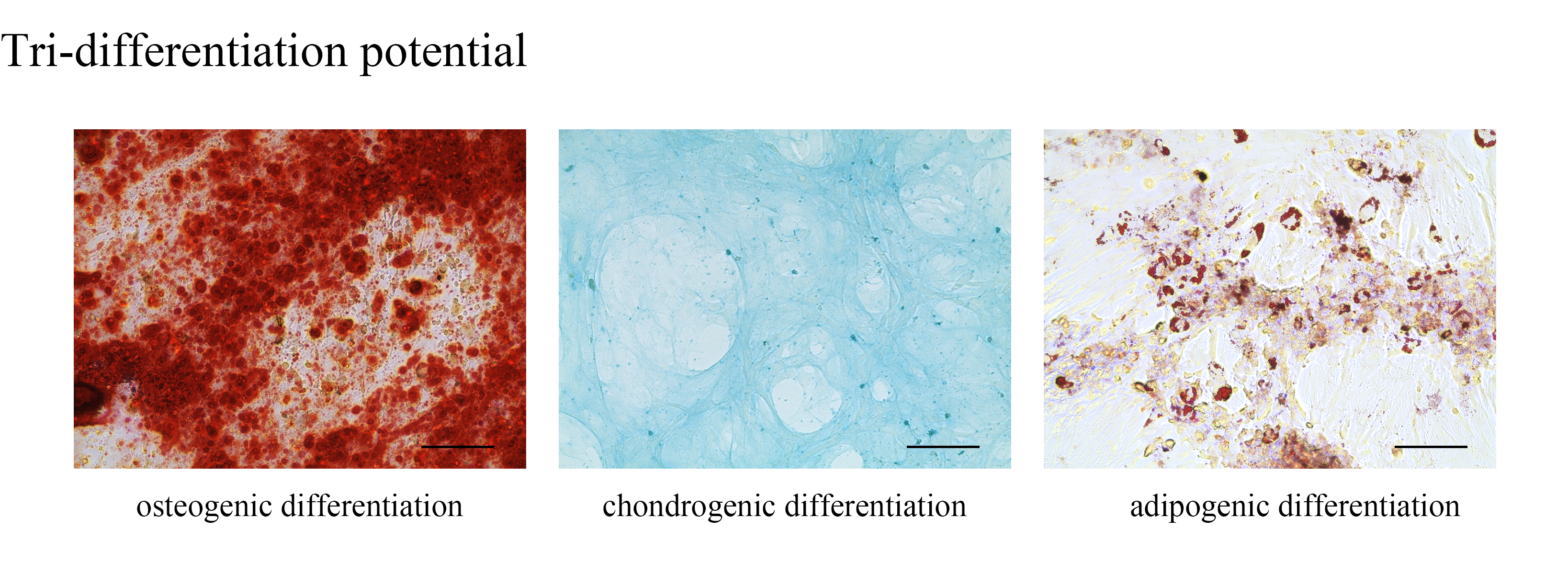

Supplement: Supplementary file 2 — Additional file 2. After 3 weeks induction of osteogenic, adipogenic and chondrogenic inductive medium, cells were stained with Alizarin Red S, Oil Red O and Alcian Blue, respectively. Scale bar = 100 μm. (TIF 4893 kb) [file 13287_2019_1464_MOESM2_ESM.tif]
